# The impact of pulp disease on oral health-related quality of life in children aged 6 to 10 years

**DOI:** 10.1007/s00784-026-06770-6

**Published:** 2026-02-23

**Authors:** Patrícia Gomes Fonseca, Maria Letícia Ramos-Jorge, Bianca Oliveira de Carvalho, Maria Eliza da Consolação Soares, Izabella Barbosa Fernandes

**Affiliations:** 1 Department of Dentistry, School of Biological and Health Sciences, Federal University of the Jequitinhonha and Mucuri Valleys, Diamantina, MG Brazil; 2 Department of Dentistry of the Institute of Life Sciences at the Federal University of Juiz de Fora/Campus, Governador Valadares, MG Brazil; 3 Department of Child and Adolescent Oral Health, School of Dentistry, Federal University of Minas Gerais, Belo Horizonte, MG Brazil

**Keywords:** Cross-Sectional studies, Dental pulp exposure, Quality of life, Children

## Abstract

**Objetives:**

To investigate the prevalence of pulp involvement and its impact on the quality of life of children aged 6 to 10 years.

**Materials and methods:**

Cross-sectional study with 363 schoolchildren. Were applied to children the “Child Perceptions Questionnaire*”* (CPQ 8–10), which assesses the impact of oral health on quality of life (OHRQoL). Oral clinical examinations were performed on the children to detect exposure of the dental pulp.

**Results:**

Of the 363 children, 73 (28.2%) had at least one pulp-exposed tooth. There was an association between lower maternal schooling and the impact on OHRQoL, both for maternal schooling from 9 to 11 years of schooling (OR = 1.23; 95% CI: 1.00-1.51; *p* = 0.045) and for 8 years or less of schooling (OR = 1.26; 95% CI: 1.00-1.59; *p* = 0.045), compared to 12 years or more of maternal schooling. Children with pulp exposure were associated with an impact on OHRQoL model adjustment (OR = 1.49; 95% CI: 1.22–1.81; *p* < 0.001).

**Conclusion:**

The prevalence of pulp involvement in children aged 6 to 10 years was 28.2%. Children with pulp involvement had a worse perception of quality of life related to oral health, with an impact on emotional, social, and functional aspects, as well as limitations caused by oral problems.

**Clinical relevance:**

Epidemiological studies commonly address data on caries experience using the classic DMFT/dmft index, which records data on the average number of decayed, missing and filled teeth, but does not provide information on dental pulp exposure, which many It can sometimes have harmful consequences and can impact the quality of life related to oral health in children.

## Introduction

Pulp involvement can occur through the progression of untreated dental caries, dental trauma, or during treatment to remove deep caries lesions [1]. Therefore, pulp involvement can be detected when the tooth structure has been destroyed and the opening of the tooth’s pulp chamber is visible, or in the presence of clinical alterations such as fistulas or abscesses [2]. Studies have shown that the prevalence of deciduous teeth with pulp involvement can vary from 19.5% to 34.9% [3, 4].

When pulp exposure is not treated, this condition may worsen [5], which often manifests itself through pain, which can compromise the performance of daily activities of individuals [6]. Within this context, oral health-related quality of life (OHRQoL) involves measuring the daily impact of oral diseases on various aspects of the daily lives of affected individuals [7]. The evaluation of this construct allows for a more comprehensive understanding of an individual’s oral health since it includes the patient’s perspectives and experiences about their oral health [8, 9]. However, the report of OHRQoL of children and their families can be affected by several factors, from social aspects to clinical factors [6, 10].

Although previous studies have explored the association between the clinical consequences of unprotected caries and oral health-related quality of life (OHRQoL) [11–15], most focus on specific and limited age groups. Our study, in turn, investigates a broader age range, encompassing children aged 6 to 10 years. Furthermore, most previous research has used the DMFT/dmft index as a clinical measure to assess caries experience [11–13, 15], which differs from the present investigation that incorporated a more sensitive and comprehensive approach by using the International Caries Detection and Assessment System – ICDAS II to detect caries lesions in early and advanced stages, together with the assessment of their consequences. This information is relevant to direct the needs of dental treatments and guide the planning of oral service actions [16].

This study aimed to investigate the prevalence of pulp involvement and its impact on the quality of life of children aged 6 to 10 years.

## Methods

### Ethical considerations

This study was approved by the Human Research Ethics Committee of the School of Dentistry under protocol number 3.366.387. Parents/guardians were invited to participate in the research with their children, and those who agreed to participate signed an Informed Consent Form (ICF). This research followed the guidelines for the development of observational studies “Strengthening the Reporting of Observational Studies in Epidemiology” (STROBE) [17].

### Study design and population

A cross-sectional study was conducted with schoolchildren aged 6 to 10 years in city located in southeastern, Brazil, from August 2019 to March 2020. The city’s population in the year 2022 was 47,702 residents, including a total of 3541 schoolchildren aged five to nine years [18].

The calculation of the sample size considered a previous cross-sectional study [19], conducted in Brazil, in which the estimate of children aged 8 to 10 years (mixed dentition phase) enrolled in public schools who presented teeth with pulp involvement was 22%, the confidence interval was 95% and the standard error was 5%. A minimum sample of 264 schoolchildren is required, according to the sample size calculation performed in the “Open Source Epidemiologic Statistics for Public Health” (Openepi.com). A correction factor of 1.2 was applied to compensate for the clustering effect, adding 20% of the initial sample, considering possible losses, and the total final sample comprised 368 students with their peers.

Participant selection was performed in two stages. In the first stage, three schools in the city were selected using a random number method. In the second stage, children who met the eligibility criteria were randomly selected from each school. If the guardians of a selected child did not authorize the child’s participation, another drawing was performed to replace the child. Students aged 6 to 10 years, regularly enrolled in schools, and their parents/guardians who agreed to participate with their children in the study by signing the informed consent form were included. Children with systemic and/or mental development disorders reported by parents/guardians were excluded.

### Training & calibration

The examiner was trained and calibrated (theory and practice) according to the clinical diagnostic criteria for dental caries of the International System for the Evaluation and Detection of Dental Caries (ICDAS-II) [20]. The calibration included the clinical examination of 30 children, who were reassessed 15 days later. The performance of the exams was compared to that of an experienced researcher (gold standard) in the detection of dental caries lesions, according to the clinical criteria of ICDAS II and pufa/PUFA index [2]. Diagnostic agreement was measured by the kappa coefficient, with intra-examiner kappa = 0.78 and inter-examiner = 0.81.

### Variables collected

#### Socioeconomic data

A structured questionnaire was sent to parents/guardians to fill out the information, along with the informed consent form. Sociodemographic information about the families was collected, and the data were categorized as follows: sex (female/male), child’s age (6 to 7 years old/8 to 10 years old); mother’s schooling (12 years or more of schooling / 9 to 11 years of schooling / or 8 years or less of schooling); monthly family income (≥ to 2 minimum wages / ˂ 2 minimum wages) and number of dependents on family income (up to 3 people / 4 or more people).

#### Quality of life related to oral health

The Brazilian version of the instrument that assesses the child’s self-perception concerning the impact of the Child Perceptions Questionnaire (CPQ _8-10_) [7] was used. This questionnaire was answered by the children and consists of 25 questions that assess 4 domains: oral symptoms (5 items); functional limitations (5 items); emotional well-being (5 items); and social well-being (10 items). The questions that make up the questionnaire depict the frequency with which the events happen. The answers are quantified on a Likert scale (0 to 5 points), with the options “Never” (score 0); “Once or twice” (score 1); “Sometimes” (score 2); “Often” (score 3); and “Every day or almost every day” (score 4). The result is expressed as the sum of the scores referring to the domains, ranging from zero (absent impact on OHRQoL) to one hundred points (maximum impact on OHRQoL) [7].

#### Clinical dental examination

The clinical dental examination of the children was carried out in the school itself, in a reserved place, with the use of artificial light (Phoenix Head Torch, Nautika Rechargeable Bivolt). The teeth were previously dried and cleaned with sterile gauze and evaluated by the calibrated examiner with the use of a clinical oral mirror (Buccal mirror, n.3 Plano, Duflex). The instruments and materials were individually packaged and sterilized to avoid contamination, following biosafety standards.

Clinical dental variables were collected according to the ICDAS II criteria [20] and the pufa/PUFA index [2]. For dental caries, each tooth surface was classified according to the ICDAS II codes. The child was considered the unit of analysis and could be classified as having the absence of moderate/extensive dental caries (ICDAS codes from 0 to 2) or the presence of moderate/extensive dental caries (ICDAS codes from 3 to 6). Pulp involvement was considered when at least one of the child’s tooth indicated the occurrence of (p) pulp exposure, (u) ulceration of the oral mucosa, (f) fistula or (a) abscess.

### Statistical analysis

Statistical analyses were performed using the Statistical Package for the Social Sciences (SPSS for Windows, version 20.0, SPSS^®^ Inc., Chicago, IL, USA). Descriptive analyses of the data were performed, and the Kruskal-Wallis test was used to evaluate the associations between the independent variables and the total score and the CPQ domains 8–10. In addition, univariate and multivariate Poisson regressions were performed to verify the associations between OHRQoL and the independent variables of the study. Adjusted Poisson models were run with explanatory variables considered relevant to the theoretical model of the study. The Prevalence Ratio (PR) was estimated, considering the 95% confidence interval (95% CI), and the significant variables with a p< value of 0.05, after adjustment, were maintained.

## Results

The final sample of the study comprised 363 children. The sample loss of 1.36% was due to incomplete forms. Among the children evaluated, 200 were female, representing 55.1% of the sample, with a mean age of 7.8 years (SD = 1.2). Of the children in the sample, 73 (28,2%) had at least one tooth with pulp involvement (Table 1). Sample characteristics were presented according to the presence or absence of teeth with pulp involvement, and no significant associations were found in the bivariate analysis (Table 1).

**Table 1:** Descriptive data of the total sample of participants (*n* = 373)

Independent variables	Pulp involvement		Total	*p*-value
	Yes *n* (%)*	No *n* (%)*	*n* (%)*	
Sex				
Female	38(52.1%)	167 (55.7%)	205 (55.0%)	0,578
Male	35 (47.9%)	133 (44.3%)	168 (45.0%)	
Child’s age				
6 or 7 years	28 (38.4%)	134 (44.7%)	162 (43.4%)	0,359
8, 9 and 10 years	45 (61.6%)	166 (55.3%)	211(56.6%)	
Mother’s schooling				
8 years or less of study	22 (30.1%)	90 (30.0%)	112 (30.0%)	0,632
9 to 11 years of study	44 (60.3%)	169 (56.3%)	213 (57.1%)	
12 years or more of study	7 (9.6%)	41 (13.7%)	48 (12.9%)	
Household income				
≥ 2 x minimum wages	1 (1.4%)	11 (3.7%)	12 (3.2%)	0,474
< 2 x minimum wages	72 (98.6%)	289 (96.3%)	361 (96.8%)	
Number of people who live off the family income				
Up to 3 people	19 (26.0%)	96 (32.0%)	115 (30.8%)	0,397
4 or more people	54 (74.0%.)	204 (68.0%)	258 (69.2%)	

Chi-square test; *p* significant < 0.001*

Children with pulp involvement had a worse perception of quality of life in the domains of emotional well-being (*p* = 0.001), social well-being (*p* = 0.001), oral limitations (p ˂ 0.001), functional symptoms (p ˂ 0.001) and the total score of the CPQ 8–10 (p ˂ 0.001). The variable presence of moderate/extensive dental caries was significantly associated with a worse quality of life in the oral limitations domain (*p* = 0.016). In addition, a significant association was found between sex (*p* = 0.016) and the social well-being domain, as shown in Table 2.

**Table 2:** Distribution of the mean scores of the quality of life domains, according to the *Child perceptions questionnaire (CPQ*_*8-10*_*)*, according to the independent variables (*n* = 363)

Variables	Oral limitations		Functional limitations		Emotional well-being		Social well-being		CPQ total	
	µ	SD	µ	SD	µ	SD	µ	SD	µ	SD
Sex										
Female	5.66	3.52	2.74	2.90	1.85	2.76	1.30	2.44	11.55	8.28
Male	5.65	3.29	2.67	3.39	2.33	3.55	2.60	4.38	13.26	11.63
*p*-value	0.667		0.344		0.504		**0.016**		0.866	
Child’s age										
6 or 7 years	5.42	3.33	2.65	2.92	1.77	2.76	1.75	3.24	11.58	9.13
8, 9 and 10 years	5.84	3.47	2.75	3.27	2.29	3.40	1.98	3.70	12.86	**1** 10.51
*p*-value	0.242		0.965		0.269		0.857		0.218	
Mother’s schooling										
12 years or more of study	5.35	2.73	2.39	2.76	1.33	2.13	0.83	1.32	9.89	6.38
9 to 11 years of study	5.75	3.49	2.80	3.14	2.04	3.00	1.95	3.64	12.55	10.07
8 years or less of study	5.60	3.53	2.67	3.25	2.41	3.69	2.19	3.81	12.88	10.81
*p*-value	0.916		0.813		0.296		0.182		0.512	
Monthly family income										
≥ 2 x minimum wages	6.82	4.77	2.73	2.97	3.82	5.25	1.64	2.42	15.00	12.12
<2 x minimum wages	5.62	3.36	2.71	3.13	2.01	3.06	1.89	3.54	12.23	9.88
*p*-value	0.225		0.815		0.200		0.578		0.339	
Number of people who live off the family income										
Up to 3 people	5.51	3.29	2.69	3.32	1.85	2.98	1.49	2.97	11.54	9.89
4 or more people	5.72	3.47	2.72	3.04	2.16	3.22	2.06	3.71	12.66	9.97
*p*-value	0.639		0.785		0.524		0.297		0.351	
Pulp involvement										
No	5.27	3.19	2.39	2.85	1.79	2.88	1.67	3.24	11.13	8.91
Yes	7.18	3.81	3.97	3.81	3.15	3.86	2.73	4.36	17.03	12.28
*p*-value	***p *** **˂ 0.001**		***p *** **˂ 0.001**		**0.001**		**0.001**		***p *** **˂ 0.001**	
Presence of moderate/severe caries										
No	4.97	3.22	2.48	2.71	1.71	2.80	1.44	2.57	10.61	8.23
Yes	5.93	3.45	2.80	3.27	2.21	3.27	2.06	3.81	13.00	10.50
*p*-value	**0.016**		0.643		0.236		0.388		0.089	

µ = Average; SD = Standard deviation; Teste de Kruskal-Wallis

The unadjusted and adjusted regression models for assessing the associations of independent variables and the negative impact of oral health on the quality of life of children and their families are described in Table 3.

**Table 3:** Unadjusted and adjusted regression models for association between independent variables and the impact of oral health on the quality of life of children and their families" (*n* = 363)

Variables	CPQ ScoreMean (SD)	Unadjusted PR CI (95%)	p* value	Adjusted PR CI (95%)	p** value
Sex					
Female	11.55 (8.28)	1			
Male	13.26 (11.63)	1.13 (0.95-1.33)	0.157		NS
Child's age					
6 or 7 years	11.58 (9.13)	1		1	
8, 9 and 10 years	12.86 (10.51)	1.08 (1.01-1.15)	0.034	1.07 (1.00-1.14)	0.061
Mother's schooling					
12 years or more of study	9.89 (6.38)	1			
9 to 11 years of study	12.55 (10.07)	1.27 (1.02-1.57)	0.030	1.23(1.00-1.51)	0.045
8 years or less of study	12.88 (10.81)	1.30 (1.02-1.66)	0.032	1.26 (1.00-1.59)	0.045
Monthly family income					
≥ 2 x minimum wages	15 (12.12)	1			
<2 x minimum wages	12.23 (9.88)	0.79 (0.52-1.20)	0.271		NS
Number of peoplewho live off the family income					
Up to 3 people	11.54 (9.89)	1			
4 or more people	12.66 (9.97)	1.05 (1.00-1.10)	0.064		NS
Pulp involvement					
No	11.13 (8.91)	1			
Yes	17.03 (12.28)	1.55 (1.29-1.87)	< 0.001	1.49 (1.22-1.81)	< 0.001
Presence of moderate/severe caries					
No	10.61 (8.23)	1		1	
Yes	13.00 (10.50)	1.23 (1.03-1.46)	0.025	1.07 (0.89-1.28)	0.499

PR= Prevalence ratio; CI= Confidence interval; NS= Not significant; SD= Standard deviation; Test: Multivariate Poisson Regression

The results of the unadjusted regression showed significant associations between the variables child age (OR = 1.08; 95% CI: (1.01–1.15); *p* = 0.034), both for maternal schooling from 9 to 11 years of schooling (OR = 1.23; 95% CI: 1.00-1.51; *p* = 0.045), and for 8 years or less of schooling (OR = 1.26; 95% CI: 1.00-1.59; *p* = 0.045), compared to 12 years or more. In addition, the variables pulp involvement (OR = 1.55; 95% CI: (1.29–1.87); *p* < 0.001) and presence of moderate/extensive caries (OR = 1.23; 95% CI: (1.03–1.46); *p* = 0.025) were also significantly associated with the impact on OHRQoL (Table 3).

In the adjusted regression, it was possible to verify that there was a significant association between the variables lower level of maternal schooling and impact on children’s OHRQoL for maternal schooling from 9 to 11 years (OR = 1.23; 95% CI: 1.00-1.51; *p* = 0.045), and for 8 or less years of schooling (OR = 1.26; 95% CI: 1.00-1.59; *p* = 0.045) compared to 12 years or more. Also, the variable presence of pulp involvement had a significant impact on children’s OHRQoL (OR = 1.49; 95% CI: 1.22–1.81; *p* < 0.001) (Table 3). This analysis was adjusted for the variables: sex, child’s age, mother’s schooling, monthly family income, number of people who live off the family income, pulp involvement and presence of moderate/severe caries. Children with pulp involvement exhibited a mean CPQ score of 17.03 (SD = 12.28), whereas those with moderate to severe caries demonstrated a mean CPQ score of 13.00 (SD = 10.50).

## Discussion

The present study evaluated the prevalence of pulp involvement and its impact on the quality of life of children aged 6 to 10 years. According to the observed data, it was detected in this study that the children evaluated had a considerable prevalence of teeth with pulp involvement. The results of this study rejected the null hypothesis tested, and the presence of pulp involvement negatively impacted the quality of life of children aged 6 to 10 years.

The prevalence of pulp involvement of 20.1% found in the present study is similar to a previous study [18] in which a prevalence of 22% was detected in Brazilian children aged 8 to 10 years. The pufa/PUFA index, which was applied in this study, reflects both the prevalence and severity of pulpo periapical diseases resulting from the evolution of dental caries [2]. Thus, these results show that a considerable portion of this population will require complex dental treatments [21], which require longer care time and represent greater trauma for the child, in addition to increased costs for caregivers. This points to the importance of adopting prevention strategies in dentistry aimed at reducing dental caries and its evolution, in order to achieve good oral health in children and avoid the worsening of this condition [22].

This research sought to expand knowledge about the impact of oral health on the quality of life of children and their families. The negative influence of the presence of dental caries on the quality of life of this population is well established in the literature [23]. In the present study, the presence of moderate/severe caries was associated with OHRQoL in the unadjusted analysis but lost its association after adjustment, while the presence of pulp involvement remained associated. This was probably due to the fact that pulp involvement is more important for altering OHRQoL than the presence of moderate/extensive caries. This can be explained by the fact that pulp involvement is a clinical consequence of untreated caries and is usually associated with pain, the evolution of infection, and can interfere with the child’s performance of basic activities with greater intensity [24]. When the CPQ 8–10 domains were evaluated individually, it was observed that only the oral limitations domain was associated with moderate/severe dental caries. In contrast, all domains (oral limitations, functional well-being, emotional well-being, social well-being) were associated with the presence of pulp involvement. The literature corroborates these findings, showing that the evolution of untreated caries can have several consequences on children’s quality of life, and this tends to worsen as the disease progresses, causing an even greater impact on OHRQoL [23, 25].

There was also an association between lower levels of maternal schooling, compared to a higher level of education, and the impact of oral health on the quality of life of the children evaluated. These results are confirmed in the literature [26, 27] and this association can be explained by considering that low schooling may represent less access to knowledge of relevant health information, such as preventive care with diet, oral hygiene habits and use of health services [28, 29]. In other words, a higher level of education and/or guidance from parents or guardians regarding the prevention of oral disorders can favor the child’s OHRQoL [27].

On the other hand, there was no association between the variable family income and OHRQoL, probably due to the homogeneity of the sample of this study in relation to this variable. In this study, it was observed that the sample was composed mostly of low-income families (monthly income less than or equal to two minimum wages) and students who study in public schools.

The variables sex and age of the child were not associated with OHRQoL in the adjusted analysis, although the child’s sex was associated with the social well-being domain and the child’s age was associated with the total CPQ score of 8–10 in the unadjusted regression. The clinical variable “patients with pulp exposure” is probably more important for altering OHRQoL than these demographic variables. On the other hand, previous studies [14, 26] have demonstrated these associations. It has been reported that females commonly have a greater concern with oral health and greater self-criticism associated with dental appearance compared to males [30]. On the other hand, the association with older age is explained by a longer time of exposure of the teeth to cariogenic challenges and, consequently, a higher risk of developing dental caries and its consequences [31].

By using the ICDAS II in combination with the PUFA/pufa index to assess dental caries and its clinical consequences, it is possible to have a more sensitive and comprehensive assessment of the stage and severity of the disease. This methodological approach allows a more detailed understanding of the impact of caries on children’s oral health-related quality of life (OHRQoL). Furthermore, by including children aged 6 to 10 years, the age range of the sample is expanded compared to previous studies that focused on more restricted age groups, such as 8–10 years [12], only 12 years [13] or preschoolers aged 4–5 years [15]. This inclusion allows a more comprehensive analysis of the variations in the impact of caries and its complications on OHRQoL throughout child development, considering both mixed dentition and changing psychosocial aspects.

Among the limitations of this study, although the questionnaire used is validated and adapted for the age group assessed, future research may benefit from the adoption of complementary methods, such as interviews with guardians, which contribute to a broader and more in-depth understanding of oral health-related quality of life in childhood (OHRQoL). Although the sampling process was random and conducted with methodological rigor, selection was restricted to public schools in a single city in the Southeast region of Brazil, which may limit the generalization of the findings to other child populations with different sociodemographic and regional contexts. Furthermore, this study presents a cross-sectional design, which precludes the establishment of causal relationships [32]. Future intervention studies are encouraged to assess the impact of treating teeth with pulp involvement on improving the quality of life of affected children.

The sample of children in this study showed a high prevalence of caries lesions and pulp involvement. There is a need to expand public health planning actions and services aimed at this age group to prevent these oral alterations and promote the oral health of this population, providing a better quality of life to these children and, at the same time, avoiding the need for increasingly complex dental treatments, which weaken the structure of the tooth [33].

## Conclusions

The prevalence of pulp involvement in children aged 6 to 10 years was 28.2%. Children with pulp involvement had a worse perception of quality of life related to oral health, with an impact on emotional, social, and functional aspects, as well as limitations caused by oral problems.

## Data Availability

Data will be made available upon request.
